# Efficacy of Behavioural Intervention, Antipsychotics, and Alpha Agonists in the Treatment of Tics Disorder in Tourette’s Syndrome

**DOI:** 10.7759/cureus.22449

**Published:** 2022-02-21

**Authors:** Muneeba Rizwan, Noor ul ain Shahid, Noreen Naguit, Rakesh Jakkoju, Sadia Laeeq, Tiba Reghefaoui, Hafsa Zahoor, Ji Hyun Yook, Lubna Mohammed

**Affiliations:** 1 Department of Research, California Institute of Behavioral Neurosciences & Psychology, Fairfield, USA

**Keywords:** alpha agonists, antipsychotics, movement disorders, non-pharmacological treatment, pharmacological treatment, pharmacotherapy, behavioural interventions, tics disorder, behavioural therapy, tourette's syndrome

## Abstract

Tourette's Syndrome (TS), in which patients have sudden, repeated, involuntary twitches and movements, called tics, is a condition of the nervous system. They can be motor, vocal, simple, or complex tics. It can be physically, emotionally, mentally, and socially distressing and challenging for those suffering from it. Usually, it is accompanied by various comorbidities like attention-deficit hyperactivity disorder, obsessive-compulsive disorder, and sleep disorders. A variety of environmental and genetic factors are also associated with tics in TS like the first-degree relatives are more at risk of developing TS.TS is heterogeneous with complicated patterns of inheritance and phenotypic manifestations. There is a strong association between common single nucleotide polymorphisms (SNP, s) in the SLITRK1 gene and TS. Environmental factors like prenatal, postnatal, and perinatal factors directly influence tics in TS. These factors are low birth weight, intrauterine growth retardation (IGR), and various infections. The treatment of TS can be broadly classified into non-pharmacological and pharmacological treatment. Non-pharmacological therapy includes various behavioural interventions that can be helpful in situations when patients are tolerant of medical treatments. Psychoeducation and counselling play an essential role in the treatment of TS. It is vital to give a proper understanding to the patient and their family about the disease. Cognitive-behavioral intervention for tics, cognitive-behavioral therapy, exposure and response prevention, relaxation techniques, deep brain stimulation, and habit reversal training are the commonly used therapies for tics. These therapies have shown good efficacy because it improves the Yale Global Tic Severity Scale score (YGTSS) significantly. And they show effectiveness in patients who are irresponsive to medical treatment. The main lines of medical treatment are antipsychotics and alpha agonists. Typical (haloperidol, pimozide) or atypical (aripiprazole, risperidone, olanzapine) Antipsychotics differ in their side effects, efficacy, and tolerance in different age groups of children. Haloperidol was the first drug approved by the Food and Drug Administration for tics, but later on, new developments and improvements were made as far as drug therapy is concerned. The alpha-agonist most commonly used is clonidine which is also available in the form of adhesive patches. Another alpha agonist which is also widely used is guanfacine. Botulinum toxin and baclofen have also shown efficacy in dealing with tics in TS with other comorbidities. We will review in this article all the main lines of treatment and their effectiveness in TS.

## Introduction and background

The prevalence of Tourette's syndrome is three to nine thousand in children who are not school-going. This syndrome is common in children, and the severity is at its peak at the age of ten to twelve years [1]. Gilles de la Tourette was the first neurologist who narrated Tourette's syndrome in 1885, and it is characterized by tics that are immediate repeating motor and vocal movements without any pattern [2]. Tourette's syndrome is associated with different comorbidities like attention deficit hyperactivity disorder (ADHD), obsessive-compulsive disorder (OCD), anxiety, mood disorders, and sleep disorders that influence the daily activities of people suffering from it [3].

The aetiology of TS is an aberration in the sensory and motor component of the corticostriatal -thalamocortical circuit and the limbic system. The dopaminergic transmission is abnormal, and that's the reason dopaminergic antagonists can reduce tics. First-degree relatives of a patient with TS have a 5% to 15% chance of getting this disease and a 10% to 20% chance of developing any form of tics. There are various genetic and environmental factors associated with Tourette's syndrome. The different environmental factors linked to this disorder can be prenatal (smoking during pregnancy, low birth weight, IGR), perinatal (perinatal hypoxia, premature death), and postnatal (different infections and psychological stress) [4]. TS is a heterogeneous disorder with complicated inheritance patterns and phenotypic manifestations. There is a strong association between Tourette's syndrome and common single nucleotide polymorphisms (SNP, s) in the SLITRK1 gene [5]. 

The main lines of pharmacological treatment for TS are alpha agonists and antipsychotics, alpha agonists being the top line of treatment for people suffering from TS with ADHD, and antipsychotics being the most efficient line of therapy [6]. Behavioural therapy is a standard non-pharmacological treatment for tics nowadays, and the most prevalent behavioural therapy is habit reversal therapy [7]. As far as the drug therapy is concerned, commonly used alpha agonists are clonidine and guanfacine, and the atypical antipsychotics commonly used are risperidone and aripiprazole [7]. The non-pharmacological treatment also includes giving knowledge to the patient about TS. Comprehensive behavioural intervention for tics (CBIT) involves coaching the patients to adopt a behaviour to ignore a tic whenever they encounter an indicative sensation [8]. Habit reversal therapy (HRT) is the commonly used behavioural therapy for tics nowadays. Exposure and response prevention (ERP) is one of the approaches used to deal with tics. Patients are advised to endure premonitory impulses for more extended periods while combating tics onset, which decreases the number of tics [9]. 

As far as pharmacological therapy is concerned, clonidine, an alpha agonist, is useful in treating Tourette's syndrome. Clonidine transdermal patch has no peak plasma concentration, which is used continuously for a week to have maximum efficacy and fewer side effects [10]. Antipsychotics such as aripiprazole improve tics in patients suffering from TS with OCD [11].

However, future researchers should be aware of how treatments available for Tourette's syndrome affect the life span of people suffering from it and which treatments should be used with minimum side effects. Also, neurologists should apply various techniques for better compliance and treatments that minimize the chances of relapse of tics in Tourette's syndrome. This study looks for different treatment options available for dealing with Tourette's syndrome and the efficacy of non-pharmacological and pharmacological lines of treatment available for tics in Tourette's syndrome. 

## Review

**Figure 1:**
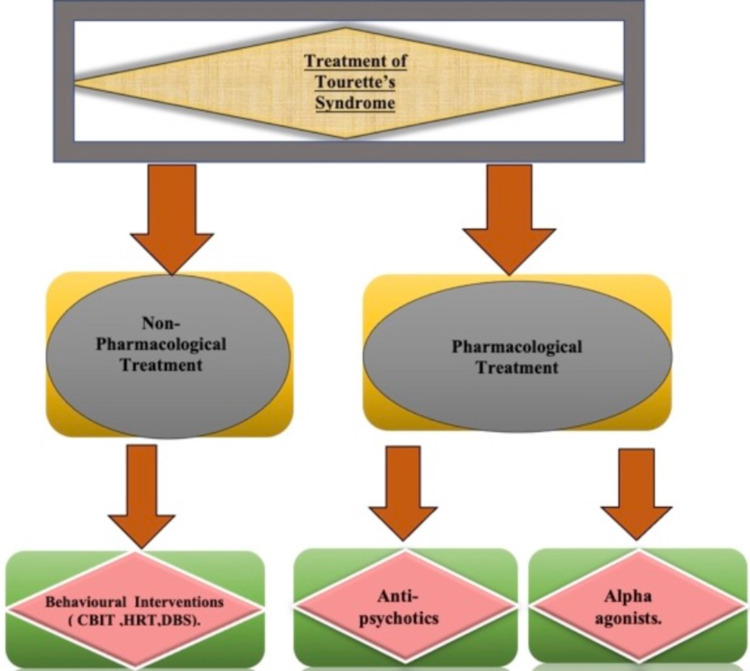
Treatment of Tourette’s syndrome The two main lines of treatment is non-pharmacological and pharmacological. The non-pharmacological treatment is psychoeducation and behavioural intervention, which mainly includes comprehensive behavioural intervention for tics (CBIT), habit reversal training (HRT) and deep brain stimulation (DBS). The pharmacological treatment includes mainly antipsychotics (Aripiprazole, Risperidone, Haloperidol, Pimozide) and Alpha agonists (Clonidine, Guanfacine), Baclofen and Botulinum toxins are also used in some cases for the treatment of Tourette’s syndrome. The idea of the figure has been adopted from the articles "Tourette syndrome: A mini-review" and "A review of the current treatment of Tourette syndrome" [1,2].

TS is seen in children, and it is most severe at the age of 10 to 12 years [1]. Tourette's syndrome is found in about two to three per thousand children aged six to 17 years. The prevalence of Tourette's syndrome is three times more in males than in females [2]. This article will include a detailed discussion of the different treatment options available for Tourette's syndrome and their efficacy. The main treatment options for TS are mainly non-pharmacological and pharmacological therapies. The primary non-pharmacological therapies are various types of behavioural interventions like CBIT, HRT, and deep brain stimulation (DBS). The foremost pharmacological agents that are primarily used are alpha agonists and antipsychotics [1,2]. The various treatment options for Tourette's syndrome are illustrated in Figure 1.

Efficacy of behavioural intervention

The non-pharmacological treatment is an option for those patients who are irresponsive to the pharmacological treatment for Tourette's syndrome. It can save the patients from the adverse effects of drug therapy. The two primary non -pharmacological approaches to treating Tourette's syndrome are deep brain stimulation (DBS) and behavioural therapy. The immediate behavioural treatment is the comprehensive behavioural intervention for tics (CBIT). 

In DBS, an electrode is placed into the brain allowing electrical activity and amend functions in the brain [2]. Multiple brain parts are targeted for subduing tics in Tourette's syndrome during DBS. YGTSS total score shows improvement when anterior globus pallidus is targeted, followed by the centromedian thalamic region and posterior globus pallidus. Due to DBS, many stimulation-induced harmful effects were seen, including dysarthria in 6.3% and paraesthesia in 8.2% of the cohort in a study conducted on one hundred and eighty-five patients with bilateral Tourette's syndrome [12].

To better understand TS, it's pivotal for all recently diagnosed patients and their families to be educated about this syndrome. They should be given all the necessary information regarding this. CBIT has issued a "high confidence" recommendation in clinical practice guidelines provided by the American Academy of Neurology (AAN) program. Habit reversal training, relaxation training, and functional interventions are designed to deal with circumstances that may aggravate tics [8]. HRT consists of nine different techniques within four discrete domains: (1) awareness training techniques to help patients recognize situations and initial danger signs related to delaying tics behaviours. (2) Competing for response technique guiding individuals to do a movement contradictory with a tic using antagonists' muscles. (3) Motivation techniques that emphasize social and environmental results of tics. (4) Generalization includes a full-fledged practice of the HRT protocol and its transition to daily life. In another technique called exposure response prevention (ERP), patients are asked to tolerate the indicative impulses between impeding tics, and these urges will result in reducing the tics [9].

So, in some specific cases of tics, when patients are tolerant to drug treatment in Tourette's syndrome, behavioural interventions can be an efficient treatment as it improves the YGTSS total score, which is an essential criterion for tics assessment. Various behavioural interventions have efficacy in patients with tics depending upon the age of the patients, the severity of the disease, and comorbidities associated with TS (Table 1).

**Table 1:** Efficacy of behavioural intervention DBS: deep brain stimulation, HRT: Habit reversal training, CBIT: Comprehensive behavioural intervention for tics, TS: Tourette’s syndrome.

Author	Year of Publication	Purpose of Study	Intervention studied	Conclusion
Seideman MF et al. [2]	2020	To discuss TS symptoms and possible causes and non-pharmacological and pharmacological treatment options nowadays.	Pharmacological options like alpha 2 Adrenergic agents (Clonidine, Guanfacine), Antipsychotics (Haloperidol, Pimozide, Aripiprazole, Risperidone, Botulinum Toxin A, Cannabinoids and other miscellaneous drugs. Non-pharmacological options like DBS, behavioural therapy, and comprehensive behavioural intervention.	The pharmacological and non-pharmacological treatment choice must be customized based on the gravity of symptoms, situations, side effects, and response to previous treatment.
Martinez-Ramirez D et al. [12]	2018	To evaluate the efficacy and safety of DBS in a multinational cohort of patients with TS.	DBS.	DBS could be a probable surgical therapy for selected patients with TS. Many patients will get DBS across multiple targets.
Billnitzer A et al. [8]	2020	To study various treatment options available for TS to improve behavioural, psychiatric, and motor symptoms.	Patient education, CBIT, Alpha 2 agonists (Clonidine, Guanfacine, Topiramate,) Antipsychotics (Aripiprazole, Risperidone, VMAT 2 inhibitors, D1 receptor antagonists, Cannabis-based medication, Botulinum Toxin A, DBS.	The foundation of any treatment is educating the patient and a therapeutic approach that is personalized and altered to address those symptoms that are most worrying to the patients. DBS should be restrained for the more refractory cases as it has its risks.
Fründt O et al. [9]	2017	To analyse behavioural therapies for treating primary tics disorders.	Psychoanalytic and supportive psychotherapy, massed (negative particle (MP), sleep treatment, HRT, (CBIT), exposure and response prevention (ERP), cognitive-behavioural treatment (CBT), contingency management (CM), functional-based interventions (FBI), relaxation training (RT), SM awareness training, mindfulness-based stress reduction, internet-based training, and telehealth approaches, autonomic modulation, and neurofeedback.	Treatment practices should be modified to the patient's individual needs, considering the age, tics severity, and neuropsychiatric comorbidities such as ADHD and OCD. Internet-based and telehealth approaches may help in easy accessibility to behavioural treatments. New non-pharmacological therapies that focus on the conversion of autonomic symptoms or attention-based interventions can also be used to treat TS.

**Figure 2:**
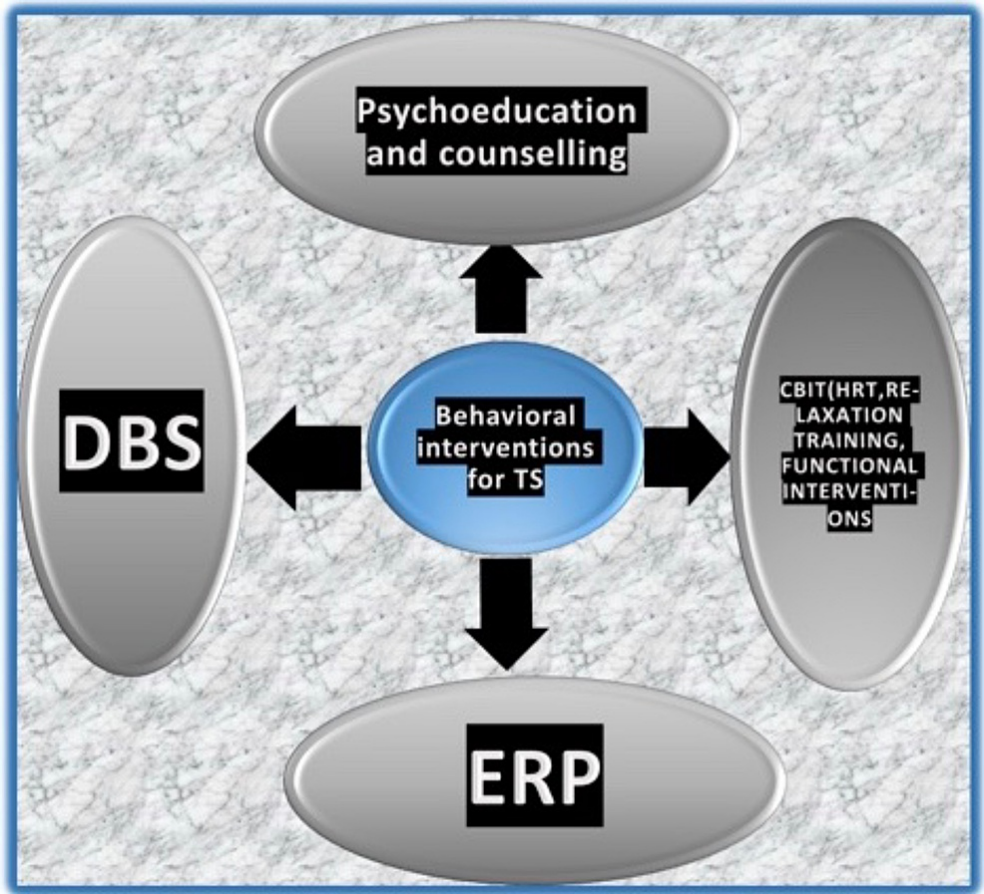
Behavioural interventions for TS mainly includes: 1) Psychoeducation and counselling 2) DBS (deep brain stimulation) 3) ERP (Exposure Response Prevention) 4) CBIT (comprehensive behavioural intervention for tics), which includes HRT (Habit Reversal Therapy), Relaxation training and different functional interventions. The idea of the figure has been adopted from the article "Tourette syndrome: A mini-review "[1].

The various behavioural interventions that are commonly practiced for the treatment of patients with TS. The foremost is psychoeducation and counselling the patient and his family about this disease. The second one is deep brain stimulation. Other interventions are exposure response prevention and comprehensive behavioural intervention for tics [1]. The various behavioural interventions for tics are shown in Figure 2. 

Efficacy of antipsychotics

Atypical Antipsychotics

Aripiprazole, which is an atypical antipsychotic, has efficacy on tics and is safe to use. Aripiprazole has a great efficacy on tics associated with obsessive-compulsive disorder. A study enrolled 133 patients from 76 practice sites in the United States, Canada, and Italy. A total of 119 patients took the study taking low doses of aripiprazole (5mg/day if <50kg ,10 mg/day if >50 kg) and high doses of aripiprazole (10 mg/day if <50 kg,20 mg /day if >50 kg) versus placebo for eight weeks. Using the YGTSS, a notable improvement in tics severity was seen with aripiprazole [2]. Aripiprazole acts on dopamine and serotonin receptors and is recommended in children with bipolar disorders and autistic disorders [13]. Aripiprazole which is an atypical antipsychotic has fewer extrapyramidal effects. That's why it is commonly used in the treatment of Schizophrenia in adults and tics in children. Aripiprazole has shown efficacy and is well tolerated in patients with TS with OCD. In a case series where six subjects were chosen who had TS with OCD, they were given aripiprazole for 12 weeks. They showed significant improvement in YGTSS (pre aripiprazole YGTSS was 31.2 whereas post aripiprazole YGTSS was 13.7), the Children's Yale-Brown Obsessive-Compulsive Scale C-YBOCS also showed improvement (pre-op aripiprazole OCD severity scale score on the C-YBOCS was 28.2, and the post aripiprazole was 8.2 [14]. 

In a randomized, double-blind, placebo-controlled trial, 34 medication-free subjects were included. After eight weeks of therapy, sixteen subjects were given risperidone; there was a 32% reduction in tics severity compared to 18 placebo patients who showed only a 7% reduction in tics severity. So, Risperidone is efficient for treating tics in TS and is safe for short-term management [15].

Olanzapine, among other antipsychotics, has a more significant activity at serotonin 5-HT2 receptors than dopamine D2 receptors and thus produces minor extrapyramidal symptoms. The most typical side effects of olanzapine are sleepiness, increase in appetite, and weight gain. A double-blind cross-over study with olanzapine versus low dose pimozide was done in which olanzapine showed decreased tics severity compared to pimozide [16].

Typical antipsychotics

Haloperidol, which is a D2 receptor antagonist, was the only drug that showed efficacy for tics. It might cause a 91% reduction in tics when given at the maximum dose. The typical antipsychotic pimozide is a dopamine receptor and calcium channel blocker. Pimozide has been associated with the prolongation of QTc interval and cardiac arrhythmias. In a double-blind, placebo-controlled, cross-over study, haloperidol had a three-fold increased risk of adverse effects than pimozide limiting its use only in severely disabled patients who are tolerant to other therapies [17]. The randomized controlled trials for the typical antipsychotics haloperidol and pimozide have shown that pimozide has more efficacy and may have more favorable adverse reactions than haloperidol [18]. FDA-approved pimozide for children > 12 years and haloperidol for use in children age > 3 years. Both haloperidol and pimozide cause extrapyramidal adverse effects like tardive dyskinesia [2].

Overall, typical antipsychotics like Haloperidol are the most efficient for severe cases of tics in TS who are irresponsive to other therapies, but its severe side effects have limited its use. It differs from other antipsychotics in respect to the side effects. Aripiprazole is an atypical antipsychotic which is well tolerated as compared to risperidone and olanzapine.

The safety and efficacy of various typical and atypical antipsychotics are shown in Table 2, although new developments are required for further improvement in the treatment of TS and better compliance in patients with TS.

**Table 2:** Efficacy of Antipsychotics DBS: Deep brain stimulation,  CDTI: Canadian disease and therapeutic index, TS: Tourette’s syndrome, OCD: Obsessive-compulsive disorder.

Author	Year of Publication	Purpose of Study	Intervention Studied	Conclusion
Seideman MF et al. [2]	2020	To discuss TS symptoms and possible causes and non-pharmacological and pharmacological treatment options nowadays.	Pharmacological options like alpha 2 Adrenergic agents (Clonidine, Guanfacine), Antipsychotics (Haloperidol, Pimozide, Aripiprazole, Risperidone, Botulinum Toxin A, Cannabinoids and other miscellaneous drugs. Non-pharmacological options like DBS, behavioural therapy, and comprehensive behavioural intervention.	The pharmacological and non-pharmacological treatment choice must be customized based on the symptoms, circumstances, side effects, and responsiveness to previous treatment.
Sallee F et al. [13]	2017	To see the efficacy and safety of tics in children and adolescents with TS.	Phase 3 was a randomized, double-blind, placebo-controlled trial with a low dose Aripiprazole, high dose Aripiprazole, low dose Aripiprazole, or placebo.	This study showed that oral Aripiprazole has safety and efficacy for tics in children and adolescents with TS.
Murphy TK et al. [14]	2004	To see the efficacy, safety, profile for Aripiprazole in GTS patients and its effect on TS and other psychiatric comorbidities (OCD, ADHD, depression), etc.	Aripiprazole.	Aripiprazole has shown efficacy and is well-tolerated in the therapy of TS and OCD.
Scahill L et al. [15]	2003	To see the efficacy and safety of risperidone in children and adults with TS.	Risperidone.	Risperidone has safety and efficacy for short-term treatment of tics in children or adults with TS. Longer-term studies are required to see the effectiveness and safety.
Roessner V et al. [16]	2011	To summarize the current unanimity on pharmacological treatment choices for TS in Europe to lead the clinician in daily practice.	Antipsychotic agents, noradrenergic agents, Nicotine, Tetrahydrocannabinol, Botulinum Toxin injections, Talipexole, Baclofen, Topiramate, Lithium, Methylphenidate, etc.	The individual therapy should be planned by considering the diagnostic information, the level of disability associated with tics, the efficacy data, side effects of treatment options, and the patient's interest for the best results and compliance.
Quezada J et al. [17]	2018	To analyse all the conventional pharmacological treatments and review those presently in development.	Alpha 2 agonists like Clonidine, Guanfacine, Baclofen, Topiramate, Botulinum Toxin A. Typical antipsychotics like Pimozide, Haloperidol, Fluphenazine. Atypical Antipsychotics like Aripiprazole, Risperidone, Olanzapine, Ziprasidone, Quetiapine, Benzamides, Tiapride. Vesicular Monoamine transporter-2 inhibitors like Tetrabenazine, Deutetrabenazine, Valbenazine, Cannabinoids, alternative agents like Ningdong Granule, and Omega 3 fatty acids.	In previous years, there has been increased interest in non-neuroleptic, noradrenergic options to control tics. Many new developments may give unique therapeutic options in treating TS.
Cothros N et al. [18]	2019	In the present study, data were chosen from the CDTI and used to interpret prescribing trends for children with tic disorders considering the class of the drug selected, the molecule, and the patient's age. These were compared with current guidelines for the treatment of tics disorders in children. The objective was to measure how closely the trends approximate current guidelines.	Alpha agonists, Antipsychotics.	Medication recommendation trends in Canada for children with tics disorders are according to the evidence-based guidelines, with reasonable evidence for increasing the usage of Alpha 2 adrenergic agonists. Future studies show that CDTI may be used as a tool for the monitoring of patients with TS.

Efficacy of alpha agonists

Generally, noradrenergic agents like clonidine, guanfacine, atomoxetine are commonly used in children, with the combination of ADHD and mild tics. For the last three decades, clonidine has been a treatment for TS. It is more widely used in America than in Europe [16]. In their randomized control trial (RCT), the syndrome study group reported drowsiness as a common side effect when using clonidine as a therapy for ADHD accompanied with TS; 28% showed moderate to severe sedation. The other side effects are dizziness, fatigue, insomnia, night terrors, hypotension, chest discomfort, and headache [18]. The leading site of action of clonidine is locus coeruleus. The clonidine adhesive patch is a transdermal therapeutic system (TTS), releasing clonidine at a relatively stable rate for a week without changes in peak plasma concentrations. Compliance of the patients is high because it can be easily used by the patients themselves as it is easy to administer, disposal is simple, administered only once a week, and quick onset of action. A study conducted on 437 patients between six and 18 years had different tics disorders, and they were divided into an active group and a control group. After four weeks of treatment, the active treatment group showed a better therapeutic response than the control group, so the clonidine adhesive patch is safe and well-tolerated by TS patients [19]. 

Guanfacine is a phenyl acetyl guanidine derivative that acts as a selective agonist of central alpha 2 adrenergic receptors; it has been used to treat children with behavioural problems, tics, sleep disorders, Tourette's syndrome, opioid withdrawal syndrome, and nicotine dependence [20]. Compared to clonidine, guanfacine produces less sedation and hypotension but is more efficient than clonidine in increasing Prefrontal cortex (PFC) working, enhancing efficacy at postsynaptic sites in PFC. Guanfacine is also commonly used to stop irrelevant motor and vocal tics in patients with Tourette’s syndrome and children with ADHD and tics who mainly cannot consume stimulant medications [21].

A double-blinded, placebo-controlled, cross-over trial showed that after four weeks of treatment with baclofen, there is a remarkable amelioration in the Clinical global impression (CGI) score and Yale Global Tic Severity Scale (YGTSS) in all subjects with no considerable adverse effects with baclofen treatment [22].

Botulinum injection has excellent efficacy in treating a variety of conditions, including enormous, unusual involuntary movements. A study conducted on 35 patients has proved that BTX injections are an effective and safe therapy for Tics in Tourette’s syndrome [23]. To summarise, patients with TS and co-morbid ADHD who are primarily young suffer more from ADHD symptoms than tics themselves, so psychological education and behavioural therapy is the choice in such cases. Still, if ADHD symptoms get worse, then medical treatment is granted chiefly to the patients. Alpha agonists are the first line of therapy [24]. Alpha agonists like clonidine and guanfacine have the greater efficacy among other noradrenergic agents, while atomoxetine helps decrease tics with co-morbidities. Antipsychotic drugs are effective for a limited time in reducing the frequency of tics. There are few apparent differences in the efficiency of these drugs on tics, but they do differ in their adverse effects [25]. The various studies depict the efficacy of different alpha agonists, other medicines, and behavioural interventions in treating TS and the comorbidities associated with them. Baclofen and botulinum toxin are also currently being used as treatment options in TS. Although, more extensive trials are still needed to improve the treatment of TS (Table 3).

**Table 3:** Efficacy of Alpha Agonists TS: Tourette’s syndrome , ADHD: Attention deficit hyperactivity disorder.

Author	Year of publication	Purpose of study	Intervention used	Conclusion
Du Y et al. [19]	2008	The study aimed to assess the therapeutic efficacy and safety of the Clonidine adhesive patch in TS.	Clonidine adhesive patch	The Clonidine adhesive patch is effective and safe for tic disorders.
Alamo C et al. [20]	2016	To study the extended-release of Guanfacine for treating ADHD.	Guanfacine	Guanfacine in treating ADHD has not been thoroughly explained. Still, there is enough experimental evidence that the stimulation of postsynaptic alpha -2A receptors are the main target of its pharmacological and therapeutic effects.
Arnsten AFT et al. [21]	2012	To discuss the history of Yale's discoveries on the neurobiology of PFC working memory functions and the identification of Guanfacine for the treatment of cognitive disorders.	Guanfacine.	The researchers at YALE depicted that it is possible to reveal the microcircuitry of cognition even at the molecular level. With the help of the expansion of the PFC in brain evolution, especially in the microcircuits of layer 3, this progress may not have occurred without this invaluable resource. More research is required to understand how genetic insults lead to changes in layer 3 microcircuits to infer new therapies for cognitive disorders.
Singer HS et al. [22]	2001	To see the efficacy of Baclofen for children with TS.	Baclofen	Baclofen has shown improvement in children with TS, although progress may be due to factors other than tics. More extensive studies which compare Baclofen against other tic-suppressing drugs are needed.
Kwak CH et al. [23]	2000	To analyse the effectiveness and safety of Botulinum toxin A (BTX) injections in patients with TS.	Botulinum Toxin	Botulinum Toxin A injections have shown efficacy and are well-tolerated therapy of tic. BTX helps in controlling the sensory and motor component of tics in TS.
Hirschtritt ME et al. [24]	2016	To discuss the symptoms, epidemiology, aetiology, comorbidities, and differential diagnosis in the treatment of TS.	Alpha 2 agonists, atypical and typical neuroleptics.	Correlation between tics and symptoms arising from TS-related comorbidities such as OCD, ADHD, and anxiety is significant for treating TS.
Hollis C et al. [25]	2016	A systematic review of the benefits and risk factors of pharmacological, behavioural, and physical therapy modalities for children and young people with TS and analyse the therapy experience of young people with TS and their parents.	Pharmacological, behavioural, physical interventions.	Antipsychotics, noradrenergic agents, and HRT/CBIT effectively reduce tics in children and young people with TS. More extensive and better-conducted trials addressing critical clinical uncertainties are required.

Limitations

This review is subject to limitations as there is no evidence of different treatment modalities affecting the life span of children and adolescents suffering from Tourette's syndrome. The other limit is insufficient data regarding compliance with non-pharmacological and pharmacological therapies for tics in Tourette's syndrome. Another limitation is that there are no particular treatments for preventing relapses in Tourette's syndrome.

## Conclusions

This review mainly focuses on the different treatment options available for TS and their efficacy. In cases where patients with tics are tolerant to medical therapy or where avoidance of side effects from drug therapy is required, various behavioural interventions like CBIT, HRT, DBS, ERT, and relaxation techniques show greater efficacy and improvement in the YGTSS total score. Antipsychotics have been commonly used as a treatment for TS since approved by FDA. Haloperidol and pimozide have been used for a long time and have shown significant efficacy. Haloperidol can cause a 91% reduction in tic severity if given at a maximum dose. But the typical antipsychotics have numerous harmful side effects that have limited their use only in severely disabled patients irresponsive to other therapies. The atypical antipsychotics like aripiprazole have good efficacy in patients with TS accompanied with comorbidities like OCD and are well-tolerated compared to other antipsychotics. Risperidone and olanzapine have shown efficacy, but they differ in the side effects they cause. Risperidone has been shown to improve the Yale Global Tic Severity Rating in TS patients. In a study conducted, olanzapine showed greater efficacy than pimozide. Alpha agonists like clonidine, guanfacine, and atomoxetine show efficacy in children with ADHD with tics. Guanfacine shows effectiveness in those patients who can consume stimulant medicines. Guanfacine causes less sedation than clonidine, which is why it is more widely used in TS. At the same time, Baclofen is used efficiently for TS as it shows improvement in CGI score and YGTSS. Also, botulinum injections are being used these days for different movement disorders, including tics, because they are safe and efficient. So, all these lines of therapy show efficacy depending upon the severity and other situations, but they differ in the side effects they cause in patients using them.
